# Decreased staging of differentiated thyroid cancer in patients with chronic lymphocytic thyroiditis

**DOI:** 10.1007/s40618-018-0882-4

**Published:** 2018-04-04

**Authors:** M. Borowczyk, A. Janicki, G. Dworacki, E. Szczepanek-Parulska, M. Danieluk, J. Barnett, M. Antonik, M. Kałużna, B. Bromińska, R. Czepczyński, M. Bączyk, K. Ziemnicka, M. Ruchała

**Affiliations:** 1 Department of Endocrinology, Metabolism and Internal Diseases, Poznan University of Medical Sciences, 49, Przybyszewskiego Street, 60-355 Poznan, Poland; 2 Department of Clinical Immunology, Poznan University of Medical Sciences, 5D, Rokietnicka Street, 60-806 Poznan, Poland

**Keywords:** Chronic lymphocytic thyroiditis, Hashimoto’s thyroiditis, Differentiated thyroid cancer, Inflammation, Cancer

## Abstract

**Purpose:**

The biological association between chronic lymphocytic thyroiditis (CLT) and differentiated thyroid cancer (DTC) has not been elucidated yet. The aim of the study was to assess whether the presence of CLT exerts any influence on clinical or histological presentation of DTC.

**Methods:**

Nine hundred and seven consecutive patients with DTC treated in the years 1998–2016 were divided into two groups according to the presence or absence of concomitant CLT. The statistical differences were analysed.

**Results:**

Out of 907 patients included in the study, 331 were diagnosed with DTC and CLT (studied group), while 576 patients with DTC but without CLT constituted a control group. The distribution of papillary and follicular thyroid cancer did not differ. In CLT group, the prevalence of pT1 was greater than for pT2–pT4 DTC (*P* = 0.0003; OR = 1.69, 95% CI 1.27–2.24) compared to controls (68.3 vs. 56.1%, respectively). The presence of multifocal lesions was similar. The thyroid capsule infiltration without extrathyroidal invasion (*P* < 0.0001; OR = 0.21, 95% CI 0.14–0.31) was more frequent in the studied group, unlike extracapsular invasion, which was significantly more often present in patients with DTC but without CLT (*P* = 0.004; OR = 1.66; 95% CI 1.17–2.34) as well as nodal involvement (*P* = 0.048; OR = 0.65, 95% CI 0.42–0.99).

**Conclusions:**

The collected data indicate a protective role of CLT in preventing the spread of the DTC. The presence of CLT might limit tumour growth to the primary site.

## Introduction

The number of patients with differentiated thyroid carcinoma (DTC), which comprises papillary thyroid carcinoma (PTC) and follicular thyroid carcinoma (FTC) with their variants, is continuously growing [1–3]. Differentiated thyroid carcinoma characterizes with the fastest growing number of new cancer diagnoses worldwide [4], with the incidence increase up to tenfold in the last 30 years [5, 6]. It is still a matter of debate whether this phenomenon may be attributed to increased morbidity or is the consequence of better diagnostics. However, the latter explanation seems to play a more important role [7].

The global increase has also been observed in the rate of diagnosis of chronic lymphocytic thyroiditis (CLT) or so-called Hashimoto’s thyroiditis, which according to International Classification of Diseases (ICD-10) characterizes with diffuse infiltration of the thyroid gland with lymphocytes, resulting in progressive destruction of the parenchyma and hypothyroidism. The incidence of CLT is now estimated to be 0.3–1.5 per 1000 individuals worldwide, making it one of the most common endocrine disorders [8, 9]. What is worth noticing, currently relatively younger patients are diagnosed, if compared to the past [10]. Moreover, CLT is the most common cause of hypothyroidism in iodine-sufficient countries [11, 12] and is also regarded the most frequent autoimmune disease [13]. Whether the relationship between an increase in the incidence of CLT and DTC is such of cause and effect remains to be elucidated.

The idea of the relationship between the chronic inflammation and cancer is not new, as the observation supporting this conclusion that leukocytes can be found in neoplastic tissue, was made already by Rudolf Virchow more than a century ago [14]. Since then, chronic esophagitis has been linked to oesophageal cancer, inflammatory bowel disease to colorectal cancer and primary biliary cirrhosis to hepatocellular carcinoma [15, 16]. Differentiated thyroid cancer is the most commonly diagnosed concomitant disease in patients with CLT referred for thyroidectomy [17], with coexistence rate ranging from 0.5 to 58% [17–20]. On the other hand, in the group of patients with PTC, the occurrence rate of CLT is 2.8 times higher compared to patients with benign thyroid diseases [21] The universal background further supports the link of the two conditions, i.e., relationship to ionizing radiation exposure and dietary iodine, as well as some molecular features, i.e., *RET/PTC* rearrangements and point mutations of the *RAS* and *BRAF* genes [20]. These observations suggest that patients with CLT might be prone towards the development of PTC [22]. Although the association between CLT and DTC is suggested since 1955 [23], it is still debatable whether CLT indeed predisposes patients to the development of DTC [24]. Moreover, data on the impact of CLT on clinical and pathological parameters of DTC are ambiguous [25]. There is a vivid discussion ongoing in the current medical literature whether the coexistence of CLT and DTC influences the course of the latter and if the patients with CLT have the more favourable impact with regards to the behaviour of diagnosed DTC. Gathered data are conflicting, while research on homogeneous population are lacking [25–27]. Many previous studies demonstrated less aggressive disease at presentation, a better outcome in patients with DTC and CLT compared to those with DTC and no CLT, or both [28–31], whereas other results did not support these conclusions [32–34].

Therefore, in our study performed on the highly homogeneous group of patients, we aimed to assess whether the presence of CLT exerts any influence on clinical or histological presentation of DTC.

## Materials and methods

We retrospectively analysed the occurrence of CLT in a cohort of patients diagnosed with DTC to determine its influence on the disease.

### Patients’ characteristics

We retrospectively analysed 907 consecutive patients treated at a single tertiary care department of endocrinology, diagnosed with DTC. The analysis covers the data collected between 1998 and 2016. According to the revised World Health Organization criteria from 2004, 837 patients had the diagnosis of PTC or its variants, while 70 patients were diagnosed with FTC or its variants [35]. The group consisted of 801 women and 106 men. All patients were Caucasians. The median age at diagnosis was 49 years, ranging from 18 to 84.

Clinical records and results of histopathological examinations of the resected specimen following thyroidectomy performed in all patients were analysed.

Patients with histopathological features suggestive of Graves’ disease or with a known previous diagnosis of Graves’ were excluded from the study.

The following data were recorded from patients’ files: gender, age at diagnosis, histological type and subtype of DTC (PTC and variants, FTC and variants), tumour size, multifocality, capsular invasion without extrathyroidal extension and histopathological staging (pTNM).

### Parameters assessed and pathological definitions

The diagnosis of CLT was confirmed by histopathological examination of the specimen from thyroidectomy, performed at the moment of DTC diagnosis and included the presence of diffuse lymphocytic infiltrate, oxyphilic cells, and the formation of primary and secondary lymphoid follicles [33]. Hashimoto’s thyroiditis, focal lymphocytic thyroiditis, and expansion of single lymphoid follicles have been discriminated. Hashimoto’s thyroiditis was defined as extensive diffuse lymphocytic infiltration (DLT) with the formation of many secondary lymphoid follicles having prominent germinal centres that are destructive to thyroid. Limited lymphocytic infiltration without alterations of the thyroid follicular cells characterized focal lymphocytic thyroiditis (FLT) [36].

**Fig. 1:**
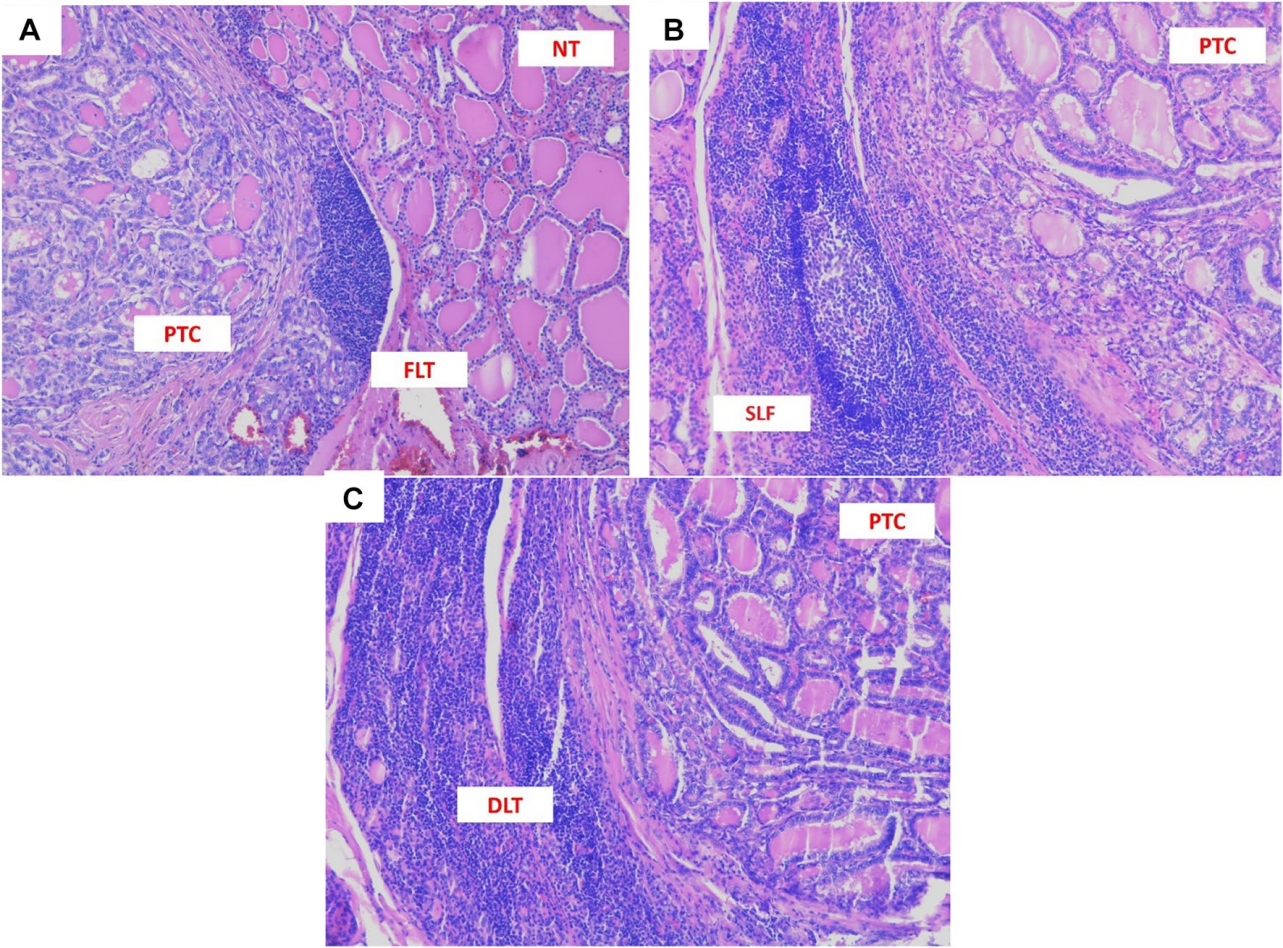
Histopathological picture of papillary thyroid carcinoma (PTC) coexisting with different types of lymphocytic infiltration at magnification 100×. **a** PTC with focal lymphocytic thyroiditis (FLT). **b** PTC with single lymphoid follicles (SLF). **c** PTC with Hashimoto’s thyroiditis (diffuse lymphocytic thyroiditis—DLT)

We also discriminated samples with the presence of single lymphoid follicles (SLF) in a mild lymphocytic inflammatory background without signs of thyroid destruction as a stage of a non-advanced immune reaction. The last two (FLT and SLF) were considered to be the less advanced thyroiditis. Figure 1 shows the examples of histopathological pictures. Tumours were supposed to be multifocal when two or more foci were found. In case of multifocality, the size of the tumour was concerned as the size of the most significant focus. The staging procedures were performed according to the American Joint Committee on Cancer TNM staging system (7th Edition) [37], as described by Girardi et al. [25]. Qualified pathologists assessed all histopathological specimens obtained during thyroidectomies.

### Statistical analysis

The parameters were recorded and entered into a dedicated database. To summarize the collected data, descriptive analysis was used. Data were tested by D’Agostino and Pearson omnibus normality test to determine the normality of continuous variables. Variables that were found to be continuous were expressed as means and respective standard deviations. Data that were found to be non-continuous were expressed as median and minimum–maximum values.

To compare differences between groups, we used the Chi-square test or Fisher’s exact test (2 × 2 contingency table), as appropriate for categorical variables. Interval data were compared with the use of the Mann–Whitney *U* test since the data did not follow normal distribution.

A *P* value of less than 0.05 was regarded as significant. Statistical analyses were performed with StatSoft Statistica v10.0 and Analyse-it for Microsoft Excel v3.53 software.

### Data availability

The datasets analysed during the current study are available from the corresponding author on reasonable request.

## Results

A group of 907 patients diagnosed with differentiated thyroid cancer were included in the study. The analysis of histopathological assessment of the specimens from thyroidectomies allowed for the discrimination between patients with DTC and concomitant CLT (*n* = 331) and the control group of patients with DTC but without CLT (*n* = 576).

Two hundred and seven patients fulfilled the criteria of Hashimoto’s thyroiditis diagnosis (62.5% of all CLT), 69 had single lymphoid follicles (SLF) (20.8%), 37 presented focal lymphocytic thyroiditis (11.2%), and 18 (5.5%) had both FLT and SLF.

Patients in the study group, with DTC and CLT, differed significantly concerning gender distribution and age at diagnosis in comparison with control group. They were younger at diagnosis (median age: 45 vs. 50 years) and female:male ratio was much higher (16.4 vs. 5.5 respectively). Patients’ characteristics are presented in Table 1.

**Table 1:** Patients’ characteristics

Characteristic	Patients with DTC and CLT *n* = 331	Patients with DTC and without CLT *n* = 576	*P* value	OR (Odds ratio) with 95% CI (confidence interval)
Female/Male, *n* (%)	312/19 (94/6)	489/87 (85/15)	< *0.0001*	*2.92* (*1.74–4.90*)
Median age at diagnosis, years (range)	45 (18–79)	50 (18–84)	<* 0.0001*	–
Multifocality, *n* (%)	68 (20.5)	89 (15.5)	0.0557	1.41 (1.00–2.01)
Capsule invasion, *n* (%)	66 (19.9)	83 (14.4)	*0.033*	*1.48* (*1.04–2.11*)
Extracapsular invasion, *n* (%)	55 (16.6)	143 (24.8)	*0.004*	*0.60* (*0.43–0.85*)
Nodal (N) involvement, *n* (%)	32 (9.7)	81 (14.1)	*0.048*	*0.65* (*0.42–0.99*)
Mean tumour size, mm (range)	15.7 (1–250)	12.1 (1–95)	0.618	–
Tumour diameter ≤ 10 mm, *n* (%)	180 (54.4)	305 (53.0)	0.285	1.06 (0.81–1.39)
Papillary thyroid carcinoma, *n* (%)	307 (92.7)	530 (92.0)	0.796	1.11 (0.66–1.85)
Classical, *n* (%)	233 (70.4)	414 (71.9)	0.703	0.93 (0.69–1.25)
Follicular, *n* (%)	50 (15.1)	80 (13.9)	0.694	1.10 (0.75–1.62)
Classical/follicular, *n* (%)	11 (3.3)	19 (3.3)	1.000	1.01 (0.47–2.14)
Oxyphilic, *n* (%)	9 (2.7)	15 (2.6)	1.000	1.05 (0.45–2.42)
Follicular/oxyphilic, *n* (%)	4 (1.2)	2 (0.3)	0.199	3.51 (0.64–19.27)
Follicular thyroid carcinoma, *n* (%)	24 (7.3)	46 (8.0)	0.796	0.90 (0.54–1.50)
Classical, *n* (%)	16 (4.9)	34 (5.9)	0.583	0.71 (0.24–2.07)
Oncocytic (Hürthle cell) variant, *n* (%)	8 (2.4)	12 (2.1)		

Italics represents statistically significant values

Chronic lymphocytic thyroiditis did not affect the multifocality of the tumours. Neither their size was changed in CLT patients. However, the thyroid capsule invasion was significantly more often present in patients in the studied group in comparison with the control group (OR = 1.48, range 1.04–2.11). The less advanced forms of chronic thyroiditis (e.g., FLT or SLF) were correlated with more advanced tumour stage (Table 2).

**Table 2:** Patients’ and tumours’ characterization due to the type of thyroid inflammation

Characteristic	Focal lymphocytic thyroiditis (FLT) *n* = 37	Single lymphoid follicles without thyroid destruction (SLF) *n* = 69	Focal lymphocytic thyroiditis and SLF *n* = 18	Hashimoto’s thyroiditis *n* = 207
Female/male, *n* (%)	35/2 (95/5)	65/4 (92/6)	18/0 (100/0)	194/13 (94/6)
Median age at diagnosis, years (range)	50 (21–79)	43 (18–72)	41 (27–62)	45 (18–79)
Multifocality, *n* (%)	11 (29.7)^a,c^	8 (11.6)^a,b^	6 (33.3)^a,c^	43 (20.8)^a,b,c^
Capsule invasion, *n* (%)	9 (24.3)^a,b^	21 (30.4)^b^	2 (11.1)^a,b^	34 (16.4)^a^
Extracapsular invasion, *n* (%)	6 (16.2)^a,c^	16 (23.2)^b^	3 (16.7)^a,c^	29 (14.0)^a,c^
Mean tumour size, mm (range)	26.7 (2–250)	9.2 (1–120)	17.9 (1–90)	8.2 (1–150)
Tumour diameter ≤ 10 mm, *n* (%)	22 (59.5)	36 (52.2)	10 (55.6)	112 (54.1)
Nodal (N) involvement, *n* (%)	7 (18.9)^a^	9 (13.0)^a^	1 (5.6)^b^	15 (7.2)^b^
Papillary thyroid carcinoma, *n* (%)	36 (97.3)	64 (92.8)	17 (94.4)	190 (91.8)
Classical, *n* (%)	29 (78.4)	40 (58.0)	13 (72.2)	151 (72.9)
Follicular, *n* (%)	6 (16.2)	17 (24.6)	4 (22.2)	23 (11.1)
Classical/follicular, *n* (%)	0	3 (4.3)	0	8 (3.9)
Oxyphilic, *n* (%)	0	3 (4.3)	0	6 (2.9)
Follicular/oxyphilic, *n* (%)	1 (2.7)	1 (1.4)	0	2 (1.0)
Follicular thyroid carcinoma, *n* (%)	1 (2.7)	5 (7.2)	1 (5.6)	17 (8.2)
Classical, *n* (%)	1 (2.7)	3 (4.3)	0	11 (5.3)
Oncocytic (Hürthle Cell) Variant, *n* (%)	0	2 (2.9)	1 (5.6)	6 (2.9)
Localization in the right lobe, *n* (%)	26 (70.3)^a^	33 (47.8)^b^	10 (55.6)^a,b^	105 (50.7)^b^
Localization in the left lobe, *n* (%)	6 (16.2)^a^	28 (40.6)^b^	6 (33.3)^a,b^	85 (41.1)^b^
Localization in both lobes, *n* (%)	5 (13.5)	8 (11.6)	2 (11.1)	17 (8.2)

a,b,c-groups followed by the same letter do not differ statistically significantly

**Fig. 2:**
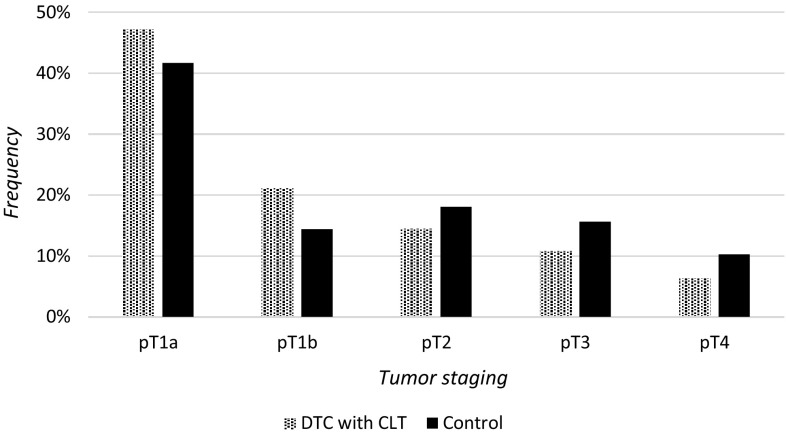
Tumour staging in analysed groups of DTC patients

No statistically significant association could be determined between any of the histopathological types and subtypes of DTC and the presence of concomitant CLT. We found that in comparison with the control group, patients with DTC and CLT presented a lower staging of thyroid cancer tumour. The prevalence of pT1 stage was greater than pT2–pT4 in the group of patients in the studied group (68.3% pT1) compared to controls (56.1% pT1) that differs statistically at *P* value = 0.0003 (OR = 1.69, 95% CI 1.27–2.24) (Fig. 2). We also found that extracapsular invasion (pT3 and pT4) was significantly more often present in patients with DTC but without CLT in comparison with patients with CLT and DTC (OR = 1.66, 95% CI 1.17–2.34, *P* = 0.004). The staging of the tumour was lower in patients with CLT (OR = 0.6, 95% CI 0.43–0.83, *P* = 0.003). Patients with CLT were also at significantly lower risk of nodal involvement (OR = 0.65, range 0.42–0.99). The risk was also higher in the less advanced forms of chronic thyroiditis.

Table 2 demonstrates patients’ and tumours’ characteristics in different types of lymphocyte infiltration.

To describe the impact of each parameter on tumour staging, we performed univariate logistic regression analysis which showed that the protective factors against higher tumour staging (pT2–pT4) were female sex, papillary thyroid cancer type (in opposition to follicular thyroid cancer) and the co-occurrence of chronic lymphocytic thyroiditis, regardless of its variant. Particular correlations are shown in Table 3.

**Table 3:** Univariate logistic regression analysis of higher tumour stage risk factors

Characteristic	Patients with pT1 DTC *n* = 549	Patients with pT2–pT4 DTC *n* = 358	*P* value	OR (Odds Ratio) with 95% CI (Confidence Interval) for lower staging
Female/Male, *n* (%)*n* (%)	496/53 (90/10)	305/53 (85/15)	*0.020*	*1.63* (*1.08–2.44*)
Median age at diagnosis, years (range)	49 (18–80)	50 (18–84)	0.817	–
Multifocality, *n* (%)	105 (19.1)	52 (14.5)	0.088	1.39 (0.97–2.00)
Capsule infiltration, *n* (%)	44 (8.0)	105 (29.3)	< *0.0001*	*0.21* (*0.14–0.31*)
Nodal (N) involvement, *n* (%)	35 (6.4)	78 (21.8)	< *0.0001*	*0.24* (*0.16–0.37*)
Papillary thyroid carcinoma, *n* (%)	529 (96.4)	308 (86.0)	< *0.0001*	*4.29* (*2.51–7.35*)
Classical, *n* (%)	413 (75.2)	234 (65.4)	0.495	1.13 (0.81–1.57)
Follicular, *n* (%)	86 (15.7)	44 (12.3)	0.489	1.17 (0.79–1.73)
Classical/follicular, *n* (%)	15 (2.7)	15 (4.2)	0.176	0.57 (0.27–1.18)
Oxyphilic, *n* (%)	13 (2.4)	12 (3.4)	0.293	0.62 (0.28–1.38)
Follicular/oxyphilic, *n* (%)	2 (0.4)	3 (0.8)	0.363	0.39 (0.06–2.32)
Follicular thyroid carcinoma, *n* (%)	20 (3.6)	50 (14.0)	< *0.0001*	*0.23* (*0.14–0.40*)
Classical, *n* (%)	13 (2.4)	36 (10.1)	0.576	0.72 (0.24–2.19)
Oncocytic (Hürthle Cell) Variant, *n* (%)	7 (1.3)	14 (3.9)		
Chronic lymphocytic thyroiditis	226 (41.2)	105 (29.3)	*0.0003*	*1.69* (*1.27–2.24*)
Hashimoto’s thyroiditis	140 (25.5)	67 (18.7)	0.808	0.92 (0.57–1.49)
Focal lymphocytic thyroiditis (FLT)	27 (4.9)	10 (2.8)	0.578	1.29 (0.60–2.77)
Single lymphoid follicles (SLT)	45 (8.2)	24 (6.7)	0.562	0.84 (0.48–1.47)
FLT and SLT	14 (2.6)	4 (1.1)	0.445	1.67 (0.54–5.20)

Italics represents statistically significant values

Multivariate logistic regression confirmed also the role of CLT in advanced tumour staging risk stratification. Logistic regression model showed that capsule infiltration and FTC increased (OR = 0.21 and OR = 0.23, *P* < 0.0001), while PTC and CLT (OR = 4.29 and OR = 1.69, *P* < 0.0001) diminished the risk of higher tumour staging similarly to the results of OR obtained by 2 × 2 contingency table.

## Discussion

The results of our study demonstrate in the largest reported to date cohort of subjects, the possible protective role of CLT in preventing the spread of the tumour by limiting tumour growth to the primary site. Both immunological and genetic mechanisms might contribute to this phenomenon.

An immunological link between DTC and CLT has been confirmed in many studies, however, their causal and temporal relationship is still not elucidated. Whether PTC develops despite immune reaction or whether CLT develops because of cross-reacting antitumour immunity, remains unclear [38].

The results of our research, as the majority of studies reported in a meta-analysis performed by Girardi et al. [25], showed a protective effect of lymphocytic thyroiditis on thyroid cancer clinical picture and prognosis [39].

In our study, the co-occurrence of CLT in DTC was found to be associated with lower tumour staging, lower risk of extracapsular invasion, and nodal involvement. Although the presence of multifocal lesions was similar in both groups, the thyroid capsule invasion without extrathyroidal extension was more frequent in patients with CLT. The findings are supported by different studies [22, 25, 40]. The collected data may indicate a protective role of CLT in the prevention of the tumour spread, while the presence of CLT might limit tumour growth to the primary site. More indolent clinical course in patients with DTC and CLT compared to those with DTC alone, including lower recurrence rates and higher overall survival rates, has already been suggested [39].

The vital role of cancer microenvironment is a subject of ongoing discussions. The protective role of CLT against the spread of DTC may be due to the participation of CD4+, CD8+, CD201+, TH17 and regulatory T cells (Treg) what was suggested by Cunha et al. [41].

Co-occurrence of CLT and PTC may also be linked with chronic stimulation of the thyroid by elevated TSH, which may initiate or promote the growth of thyroid neoplasm [42, 43].

An immunological link between CLT and DTC also lies in thyroglobulin and TgAb antibodies as it represents the primary target antigens for cellular cytotoxic and humoral immune reactions in both chronic lymphocytic thyroiditis and differentiated thyroid cancer [38].

Patients with DTC coexisting with CLT significantly differed also regarding age and gender distribution from those with DTC without CLT. They were much younger (approximately 5 years) and had a higher female predominance (over twofold). The findings remain in accordance with studies of Singh et al. [31], Loh et al. [29] and Ahn et al. [39], who reported that PTC patients with CLT were, respectively, 2, 3 or 5 years younger than those without CLT. These may raise an additional bias of the study—the finding of smaller tumours in this category could be indeed due the shorter time to progression. Previous research also confirmed that women were more prone to be diagnosed with DTC in the course of CLT [39, 44]. The latter may be linked to general female predominance in thyroid autoimmune diseases [45].

One of the factors limiting the interpretation of our findings is a selection bias, which should be taken into consideration before conclusions are derived. The observed lower staging of DTC, when concomitant with CLT, may be found due to earlier diagnosis of DTC in patients with CLT.

It has been previously suggested that the protective phenomenon of CLT in PTC stems from the immunologically derived destruction of the thyroid tissue by CLT. Kimura et al. reported that IL-1 secreted by the infiltrating lymphocytes in CLT might have exerted an anti-tumourigenic action in some cancer cells differentiation and replication, what may explain the inhibition of human thyroid cancer cell growth in patients with concomitant CLT and DTC [46].

To prevent selection bias and to limit confounding environmental factors, as a study group we have chosen mostly homogenous (4.7% non-ethnic Poles within Poland), ranking seventh in a study of 159 countries worldwide and third in Europe [2].

In conclusion, the results of our study confirm that the presence of CLT may have a significant protective role in a reduced volume of a tumour and capsule infiltration, lowering the chance for more advanced stages of DTC. The presence of advanced CLT may limit the tumour growth to the primary site. Comprehensive analysis of immunological, genetic and environmental factors, which may contribute to the observation, is required.
